# Pro-inflammatory Monocyte Phenotype and Cell-Specific Steroid Signaling Alterations in Unmedicated Patients With Major Depressive Disorder

**DOI:** 10.3389/fimmu.2018.02693

**Published:** 2018-11-23

**Authors:** Helge Hasselmann, Stefanie Gamradt, Aline Taenzer, Jan Nowacki, Rami Zain, Kostas Patas, Caren Ramien, Friedemann Paul, Katja Wingenfeld, Dominique Piber, Stefan M. Gold, Christian Otte

**Affiliations:** ^1^Klinik für Psychiatrie und Psychotherapie, Campus Benjamin Franklin, Charité - Universitätsmedizin Berlin, Berlin, Germany; ^2^Institut für Neuroimmunologie und Multiple Sklerose, (INIMS), Zentrum für Molekulare Neurobiologie, Universitätsklinikum Hamburg Eppendorf, Hamburg, Germany; ^3^NeuroCure Clinical Research Center, (NCRC), Charité - Universitätsmedizin Berlin, Berlin, Germany; ^4^Experimental and Clinical Research Center, Max Delbrueck Center for Molecular Medicine and Charité Universitätsmedizin Berlin, Berlin, Germany; ^5^Klinik für Neurologie, Charité - Universitätsmedizin Berlin, Berlin, Germany; ^6^Medizinische Klinik m.S. Psychosomatik, Charité - Universitätsmedizin Berlin, Berlin, Germany

**Keywords:** depression, monocytes, inflammation, steroid hormones, innate immunity

## Abstract

Several lines of evidence have strongly implicated inflammatory processes in the pathobiology of major depressive disorder (MDD). However, the cellular origin of inflammatory signals and their specificity remain unclear. We examined the phenotype and glucocorticoid signaling in key cell populations of the innate immune system (monocytes) vs. adaptive immunity (T cells) in a sample of 35 well-characterized, antidepressant-free patients with MDD and 35 healthy controls individually matched for age, sex, smoking status and body mass index. Monocyte and T cell phenotype was assessed by flow cytometry. Cell-specific steroid signaling was determined by mRNA expression of pre-receptor regulation (11β-hydroxysteroid dehydrogenase type 1; *11*β *-HSD1*), steroid receptor expression [glucocorticoid receptor (*GR*) and mineralocorticoid receptor (*MR*)], and the downstream target glucocorticoid-induced leucine-zipper (*GILZ*). We also collected salivary cortisol samples (8:00 a.m. and 10:00 p.m.) on two consecutive days. Patients showed a shift toward a pro-inflammatory phenotype characterized by higher frequency and higher absolute numbers of non-classical monocytes. No group differences were observed in major T cell subset frequencies and phenotype. Correspondingly, gene expression indicative of steroid resistance (i.e., lower expression of *GR* and *GILZ*) in patients with MDD was specific to monocytes and not observed in T cells. Monocyte phenotype and steroid receptor expression was not related to cortisol levels or serum levels of IL-6, IL-1β, or TNF-α. Our results thus suggest that in MDD, cells of the innate and adaptive immune system are differentially affected with shifts in monocyte subsets and lower expression of steroid signaling related genes.

## Introduction

Several independent lines of evidence have implicated the immune system in the pathobiology of mood disorders, particularly major depressive disorder (MDD) (1–3). Numerous studies and several meta-analyses have demonstrated higher levels of circulating cytokines, such as interleukin 6 (IL-6) and tumor necrosis factor α (TNF-α), in MDD (4, 5). However, serum levels of cytokines reveal little about the specific cause of immune dysfunction and the cellular source of inflammation in MDD remains poorly understood.

Importantly, it is now becoming increasingly clear that MDD is not simply a state of general immune activation but that innate and adaptive immune responses might be differentially affected (6). Considerable evidence points toward activation of monocytes in MDD (7, 8). In contrast, recent studies have suggested that adaptive immunity, specifically T cell function, might be impaired in MDD (9–11).

This implies that immune activation and impaired regulation of inflammation in MDD might be limited to certain components of the immune system. Glucocorticoids (GCs) are among the most potent endogenous regulators of inflammation, and cell-specific alterations in steroid signaling are thus promising candidates in this respect. Several studies have suggested that leukocyte responsiveness to GCs is blunted in patients with MDD (7, 8, 12–15). If regulatory pathways of inflammation (such as glucocorticoid signaling) were indeed affected in a cell-specific fashion in MDD, this might have implications for developing tailored pharmacological approaches in the future.

In the current study, we therefore aimed to explore the interplay between inflammation and stress hormone signaling by contrasting the phenotype and glucocorticoid signaling of key cell populations in the innate immune system (monocytes) vs. adaptive immunity (T cells) in a sample of well-characterized, antidepressant-free patients with MDD and closely matched healthy controls (HC). In addition, we explored the association of these putative immune signatures with serum cytokines, clinical characteristics of MDD and important risk factors such as childhood trauma.

## Materials and methods

### Participants and clinical assessments

The study was approved by the local ethics committee (EA1/096/15). The authors assert that all procedures contributing to this work comply with the ethical standards of the relevant national and institutional committees on human experimentation and with the Helsinki Declaration of 1975, as revised in 2008. All participants provided written informed consent and received financial reimbursement for their time and effort.

Patients with MDD between 18 and 60 years of age were recruited from our inpatient wards, via onsite psychiatrist referral, or online advertisements. Healthy controls were recruited from online advertisements. Patients and controls were matched pairwise on sex, smoking status, age, and body mass index (BMI) prior to running any biological analyses.

All participants were free of significant medical illness (e.g., diabetes, autoimmune or infectious illnesses), immunomodulatory treatment [e.g., non-steroidal anti-inflammatory drugs (NSAIDs), glucocorticoids or antibiotics], pregnancy, and recent (< 3 months) vaccinations. Inclusion criteria specific to MDD patients were a clinician-confirmed diagnosis of MDD, a minimum antidepressant-free period of 2 weeks and absence of comorbid psychiatric disorders (e.g., substance abuse in the past 12 months) except for mild-to-moderate anxiety disorders. Inclusion criteria specific to healthy controls were absence of any form of psychiatric illness, a Montgomery Asberg Depression Rating Scale (MADRS) score < 7 and no clinically confirmed diagnosis of any affective disorder in a first-degree relative.

Diagnosis of MDD was confirmed by experienced psychiatrists (DP, CO). In addition, during the study visit, the Mini-International Neuropsychiatric Interview (16) and the MADRS (17) was conducted by a trained clinical rater (HH). Self-report questionnaires were obtained to quantify levels of anxiety (Beck Anxiety Inventory, BAI) (18), depression severity (Beck Depression Inventory II, BDI-II) (19) and adverse childhood experience (Childhood Trauma Questionnaire, CTQ) (20).

### Blood and saliva collection

A sample of 70 ml of venous blood was collected in heparinized tubes (BD, Germany). To control for circadian rhythms and other potential confounds, samples were obtained between 8.00 a.m. and 9:30 a.m. after 12 h overnight fasting. Peripheral blood mononuclear cells (PBMCs) were immediately isolated and cryopreserved until assayed (see below for details).

Serum was collected in serum separator tubes (BD, Germany) and allowed to clot for 30 min at room temperature in the dark. Next, samples were centrifuged for 5 min, after which serum was aliquoted and stored at −20°C until analysis. Saliva samples were collected on 2 consecutive days at 8:00 a.m. and 10.00 p.m. using Sarstedt salivettes (Sarstedt, Germany) at home by the participants (within a week of their clinical visit) and shipped back to the lab in pre-stamped envelopes provided by the study team. All samples arrived within 7 days and were immediately processed and stored until assayed (see below for details).

### Isolation of peripheral blood mononuclear cells (PBMCs)

All blood samples were processed within 1 h of collection. PBMCs were isolated from heparin venous blood samples via density gradient centrifugation. In brief, samples were carefully layered on top of density medium (Biocoll, Biochrome, Germany). After centrifugation, PBMCs were harvested from the interphase, washed twice in phosphate-buffered saline (PBS) and taken up in RPMI-1640 + GlutaMax medium (Gibco, ThermoFisher Scientific, Germany) supplemented with 25% heat-inactivated fecal calf serum (FCS) (Biochrome, Germany) and 10% dimethylsulfoxide (Applichem GmbH, Germany) for cryopreservation. Cells were frozen at a concentration of 10^7^ cells/ml at −80°C in a pre-cooled freezing container. After 24–48 h, cells were transferred to liquid nitrogen and stored at −196°C until analysis.

For thawing, the cryo vials were transferred to a water bath pre-warmed to 37°C. After 5 min, 10^7^ cells were transferred into 10 ml of thawing medium (RPMI-1640 + GlutaMax containing 10% FCS at 37°C). Cells were then washed in medium, counted and prepared for phenotyping by flow cytometry or magnetic-activated cell separation (MACS Microbead Technology, Miltenyi Biotec, Germany) as described below.

### Flow cytometry

Antibody panels used for this study are presented in Table S1 (all Biolegend, UK). All monoclonal antibodies were pretested and titrated to optimal concentrations using PBMCs from healthy donors. All steps were conducted at room temperature unless otherwise specified.

First, PBMCs were incubated with a live/dead marker (Zombie NIR Fixable Viability Kit, BioLegend, UK) and the CCR7 antibody in PBS for 15 min. Next, antibody premixes were added in staining buffer (PBS + 0.5% bovine serum albumin Miltenyi Biotec, Germany + 0.02% sodium azide Sigma-Aldrich, Germany) and incubated for an additional 15 min. Cells were then washed and resuspended in staining buffer and immediately analyzed on a FACSCanto II (BD, Germany). Matched HC/MDD pairs were analyzed in the same run on the same day to avoid any systematic variation due to technical variability.

The gating strategies to identify PBMC subpopulations are depicted in detail in Figures S1, S2. Briefly, leukocyte identification followed recommendations for general immunophenotyping in humans (21, 22).

Results from manual analysis of flow cytometric data were further validated by means of an unsupervised clustering algorithm (CITRUS, as implemented in the cloud-based Cytobank software, Cytobank Inc. USA). This approach accounts for the continuous nature of immune subsets (e.g., monocyte subpopulations) and has been shown to better reflect pathophysiologic conditions than gating-based, threshold-driven manual analysis of distinct subtypes (23). Briefly, CITRUS performs hierarchical clustering of cellular populations based on phenotypical similarity and automatically identifies stratifying features between groups (24). In our analysis, we defined CD3^−^ non-T cells as input population after removing debris, doublets and dead cells. CITRUS was run with CD14, CD16, CD20, CD56, and HLA-DR as clustering channels on 10^4^ events sampled per file with a minimum cluster size threshold of 1.5%. The correlative model SAM was used to detect associations between the relative cluster abundance of each sample and the study groups (HC or MDD). Using a false discovery rate of 1%, significant clusters were exported as FCS files, concatenated for the whole study cohort and projected on viSNE maps of the total non-T cell population. The viSNE algorithm reduces multi-dimensional flow cytometry data to two dimensions (tSNE1 and tSNE2 = t-Distributed Stochastic Neighbor Embedding) while retaining the single-cell representation of the data (25).

### Cell sorting, RNA isolation, cDNA synthesis, and real-time reverse transcription-polymerase chain reaction (RT-qPCR)

For analysis of cell-specific gene expression, T cells and CD14^+^ monocytes after thawing were purified from PBMCs following manufacturer's instructions using magnetic-activated cell sorting (CD3 and CD14 MicroBeads, Miltenyi Biotec, Germany). Briefly, 10^7^ were resuspended in 80 μL MACS buffer (PBS, 0.5% BSA, and 2 mM EDTA) and 20 μL of CD3 or CD14 MicroBeads, respectively, and incubated for 15 min at 4°C in the dark. After washing with 2 mL MACS buffer per 10^7^ cells, cells were resuspended in 500 μL MACS buffer before proceeding to magnetic separation on MACS LS columns. Cell purity was checked using flow cytometry. In our hands, this yields a purity of 96.5 ± 1.2% for T cells and 92.3 ± 1.7% for CD14^+^ monocytes. RNA was isolated from purified cells using Qiagen RNeasy Plus Mini Kit (Qiagen, Germany) following manufacturer's instruction. Purity and concentration were determined using a NanoDrop spectrophotometer (NanoDrop 2000c, ThermoFisher Scientific, Germany). Average RNA yield was similar across groups for T cells [MDD: 581.1 ± 480 ng; HC: 631.9 ± 383 ng; T_(34)_ = 0.61; *p* = 0.55] and monocytes [MDD: 815 ± 1358 ng; HC: 685 ± 524.6 ng; T_(34)_ = 0.67; *p* = 0.51] All steps were conducted at room temperature. Isolated RNA was directly transcribed to complementary DNA (cDNA) without intermittent freezing using the RevertAid H Minus First Strand cDNA Synthesis Kit (ThermoFisher Scientific, Germany) according to manufacturer's instructions and stored at −80°C until analysis. cDNA was amplified on a StepOne Real-Time PCR system (Applied Bioscience, Germany) using TaqMan Gene Expression Assays (ThermoFisher Scientific, Germany) for *GR* (Hs00353740_m1), *MR* (Hs01031809_m1), *GILZ* (Hs00608272_m1), *11ß-HSD1* (Hs01547870_m1). Gene expression was normalized using two housekeeping genes: Importin 8 (*IPO8*; Hs00183533_m1) and TATA Box Binding Protein (*TBP*; Hs00427620_m1). All RT-qPCRs reactions were performed in triplicates with a patient and matching control sample on the same plate. Gene transcript levels were assessed relative to *IPO8* and *TBP* using the ΔΔCT method.

### Analysis of salivary cortisol

Circadian peak and nadir measures of hypothalamus-pituitary adrenal (HPA) axis activity were estimated by salivary cortisol levels at 8:00 a.m. and 10:00 p.m. collected on 2 consecutive days. After collection, saliva tubes were centrifuged for 5 min and aliquots stored at −20°C until analysis. Samples were analyzed in duplicates using an enzyme-linked immunosorbent assay (ELISA) (IBL, Germany) following manufacturer's instructions. Matched HC/MDD pairs were measured on the same microplate. Standard curves were fitted using 4-parameter logistics. This method has a detection sensitivity of 0.135 nmol/L and intra- and inter-assay coefficients of variation < 10%.

### Analysis of serum cytokines

Serum interleukin-6 (IL-6), interleukin 1 beta (IL-1β) and tumor necrosis factor alpha (TNF-α) levels were analyzed in duplicates using commercially available high sensitivity ELISA kits (R&D Systems Europe, UK) following manufacturer's instructions. Matched HC/MDD pairs were measured on the same microplate. Optical density was determined on a CLARIOStar microplate reader (BMG Labtech, Germany). Standard curves were fitted using 4-parameter logistics. Calculated cytokine concentrations < 0.5 x limit of quantification (LOQ, i.e. lowest standard concentration) were set to 0.5 x LOQ. The mean limit of detection for IL-6, IL-1β, and TNF- α as provided by the manufacturer is 0.031, 0.033, and 0.022 pg/mL, respectively. Intra- and inter-assay coefficients of variation were < 10%.

### Routine blood tests

Serum CRP analysis by particle-enhanced turbidimetric immunoassay (PETIA) and a differential blood cell count to enumerate circulating leukocyte subsets were conducted by a clinically licensed diagnostic lab (Labor Berlin—Charité Vivantes GmbH, Germany).

### Statistics

Cell population specific parameters were expressed as either absolute cell counts or percentages normalized to suitable reference populations. Continuous variables were analyzed with paired-sample *t*-tests due to the close matching based on four variables (age, sex, smoking, BMI, see Table S2) between patients and healthy controls (26). Dichotomous variables were analyzed with McNemar's test. Associations with clinical variables and immune markers were explored using Spearman's correlation coefficients. A two (group = MDD vs. HC) × four (time = day 1 morning vs. day 1 evening vs. day 2 morning vs. day 2 evening) repeated-measures analysis of variance (ANOVA) was run to investigate group differences in saliva cortisol levels. Effect sizes were calculated as Hedges' *g* for *t*-tests and partial eta2 (η_*p*_2) for ANOVAs (27). Statistical analyses were conducted in SPSS version 21 (IBM Inc., USA) and GraphPad Prism version 7 (GraphPad Software Inc., USA). Flow cytometry data were analyzed using FlowJo version 10.1 (Treestar Inc., USA) and Cytobank analysis software (Cytobank Inc., USA).

## Results

Demographic and clinical characteristics of patients and controls are displayed in Table 1. The majority of MDD patients were inpatients (*n* = 18) and had a recurrent disease course (*n* = 28) with a mean of 3.75 previous episodes (range: 1–7, standard deviation 1.7 episodes). Mean MADRS scores indicated moderate depression severity.

**Table 1:** Sample characteristics.

	**MDD (*n* = 35)**	**HC (*n* = 35)**	**Test statistic**	*p-*value^*^
Age, years	31.7 (11.2)	31.7 (10.2)	T_(df = 34)_ = 0	>0.99
BMI, kg/m^2^	23.9 (3.6)	23.5 (3.2)	T_(df = 34)_ = 1.27	0.21
% Females (n)	71.4 (25)	71.4 (25)	x2_(df = 1)_ = 0	>0.99
% Current smokers (n)	34.3 (12)	34.3 (12)	x2_(df = 1)_ = 0	>0.99
MADRS	24.9 (5.3)	1.2 (1.8)	T_(df = 34)_ = 26.31	< 0.01
BDI-II	29.9 (6.5)	2.9 (3.4)	T_(df = 34)_ = 21.65	< 0.01
BAI	20.7 (12.4)	3.8 (2.9)	T_(df = 34)_ = 7.41	< 0.01
CTQ Total Score	40.9 (15.9)	32.9 (9.8)	T_(df = 34)_ = 2.32	0.03
% Comorbid anxiety disorder (n)	28.6 (10)	–	–	–
% MDD subtype (n)	60 (21)	–	–	–
% Melancholic (n)	54.3 (19)	–	–	–
% Atypical (n)	5.7 (2)	–	–	–

*BAI, Beck Anxiety Inventory; BDI-II, Beck Depression Inventory II; BMI, body mass index; CTQ, Childhood Trauma Questionnaire; HC, healthy control; MADRS, Montgomery Asberg Depression Rating Scale; MDD, major depressive disorder*.

*Unless specified otherwise, values represent mean (standard deviation)*.

* *Paired-samples t-test for continuous and McNemar's test for dichotomous variables*.

As expected, the MDD group showed higher levels of anxiety and childhood trauma. There were no differences in any of the measured demographic or lifestyle variables (Table S2).

### Immune phenotype

**Figure 1:**
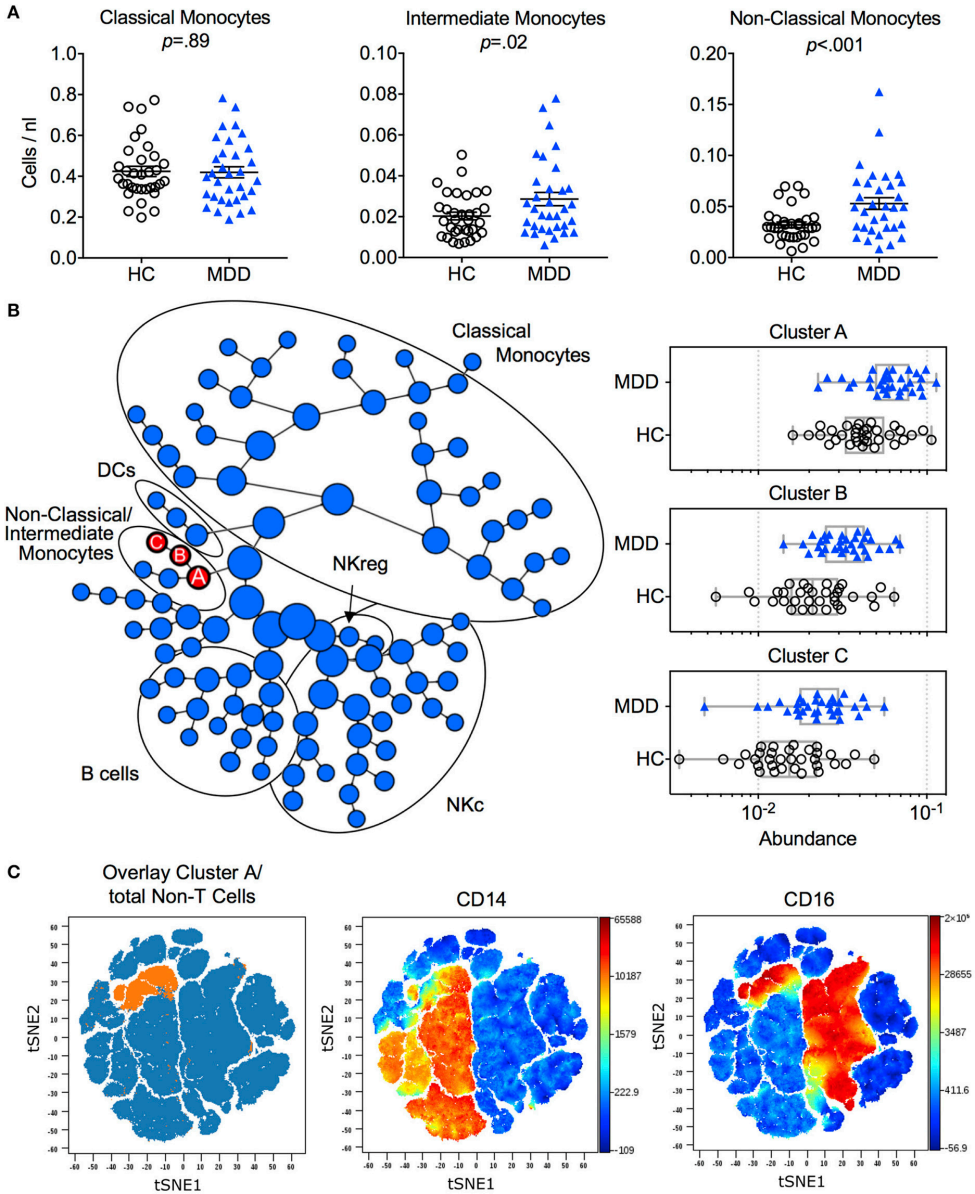
Immune phenotype in MDD patients and matched healthy controls. **(A)** Absolute cell counts of monocyte subtypes (mean ± S.E.M) in MDD patients compared to matched healthy controls. Gating strategy for identification of classical (CD14^++^/CD16^−^), intermediate (CD14^++^/CD16^+^) and non-classical (CD14^+^/CD16^++^) monocytes is depicted in Figure S1. **(B)** Results of manual gating were confirmed by means of an automated clustering algorithm (CITRUS) which identified group differences in the abundance of clusters A, B and C corresponding to monocytes expressing CD16. **(C)** In order to visualize these stratifying subsets on single cell viSNE maps, FCS files of cluster A (containing all events from clusters B and C) were exported per subject, concatenated and projected on the total input population (= Non-T cells). HC, Healthy Controls; MDD, Major Depressive Disorder; DCs, Dendritic Cells, NKc, cytotoxic NK cells, NKreg, regulatory NK cells.

When examining the phenotype of cell subsets in the innate and adaptive immune system, we observed a significantly reduced relative frequency of classical monocytes [*T*_(34)_ = 4.81; *p* < 0.0001; Hedges' *g* = 0.88] and, conversely, elevated levels of “non-classical” monocytes [*T*_(34)_ = 4.33; *p* = 0.0001; Hedges' *g* = 0.80] in MDD patients compared to controls (Figure S3B). When expressed as absolute cell counts, significantly higher numbers in MDD were only detected for non-classical and intermediate monocytes (Figure 1A). There were no statistically significant differences in circulating numbers of leukocyte subsets (Figure S3A) or relative frequencies of major lymphocyte populations (Figures S3C,D) between the groups.

To validate monocyte subset results, we applied an unsupervised clustering algorithm (CITRUS), which automatically identifies differentially abundant cell clusters between groups. This analysis yielded one major cluster with two subclusters (A, B, C) corresponding to the non-classical and intermediate monocyte cell populations (Figures 1B,C). Further confirming the results from the manual gating, no additional group differences were detected by this algorithm (Figure 1B).

### Cell-specific expression of steroid-signaling-related genes

**Figure 2:**
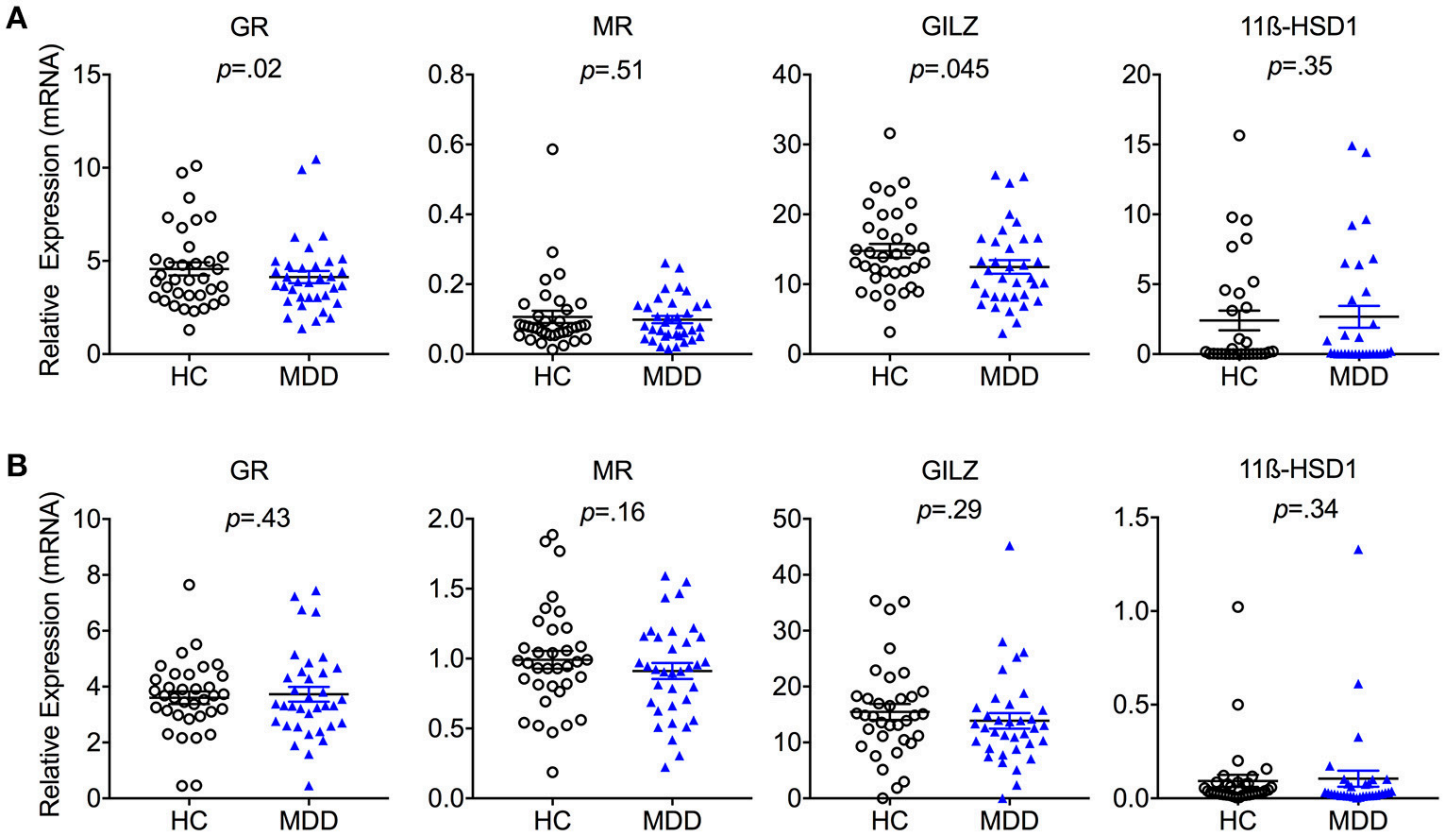
Cell-specific steroid signaling in MDD patients and matched healthy controls. Steroid-related gene expression (mean ± S.E.M) in purified **(A)** monocytes and **(B)** T cells in MDD patients compared to matched healthy controls. GR, Glucocorticoid Receptor; MR, Mineralocorticoid Receptor; 11β-HSD1, 11β-Hydroxysteroid Dehydrogenase Type 1; GILZ, Glucocorticoid-Induced Leucine Zipper Gene; HC, Healthy Controls; MDD, Major Depressive Disorder. Gene expression is depicted as fold change relative to housekeeping genes.

In a next step, we explored cell-specific alterations in glucocorticoid signaling in monocytes and T cells. Purified CD14^+^ monocytes from MDD patients expressed significantly lower mRNA levels of *GR* [*T*_(34)_ = 2.49; *p* = 0.018; Hedges' *g* = 0.21] as well as its downstream target *GILZ* [*T*_(34)_ = 2.08; *p* = 0.045; Hedges' *g* = 0.39] (Figure 2A). In contrast, no group differences in monocyte expression of *MR* or *11*β*-HSD-1* were observed (all *p*-values > 0.05). There were also no group differences in T cell expression levels of any of the genes examined (*GR, GILZ, MR*, or *11*β*-HSD-1)* (Figure 2B).

### HPA axis activity

Saliva samples were available from *n* = 30 patient/control pairs. Analysis of cortisol levels revealed no group x time interaction or main effect of group [group × time: *F*_(3;84)_ = 1.19, *p* = 0.32, η_*p*_2 = 0.04; group: *F*_(1;28)_ < 0.01, *p* = 0.93, η_*p*_2 < 0.01]. As expected, there was a main effect of time [*F*_(3, 84)_ = 36.8, *p* < 0.01; η_*p*_2 = 0.57] across groups, showing the typical circadian rhythm of HPA axis activity with higher cortisol levels after awakening compared to evening levels in both groups (Figure S4).

### Serum immune markers

**Figure 3:**
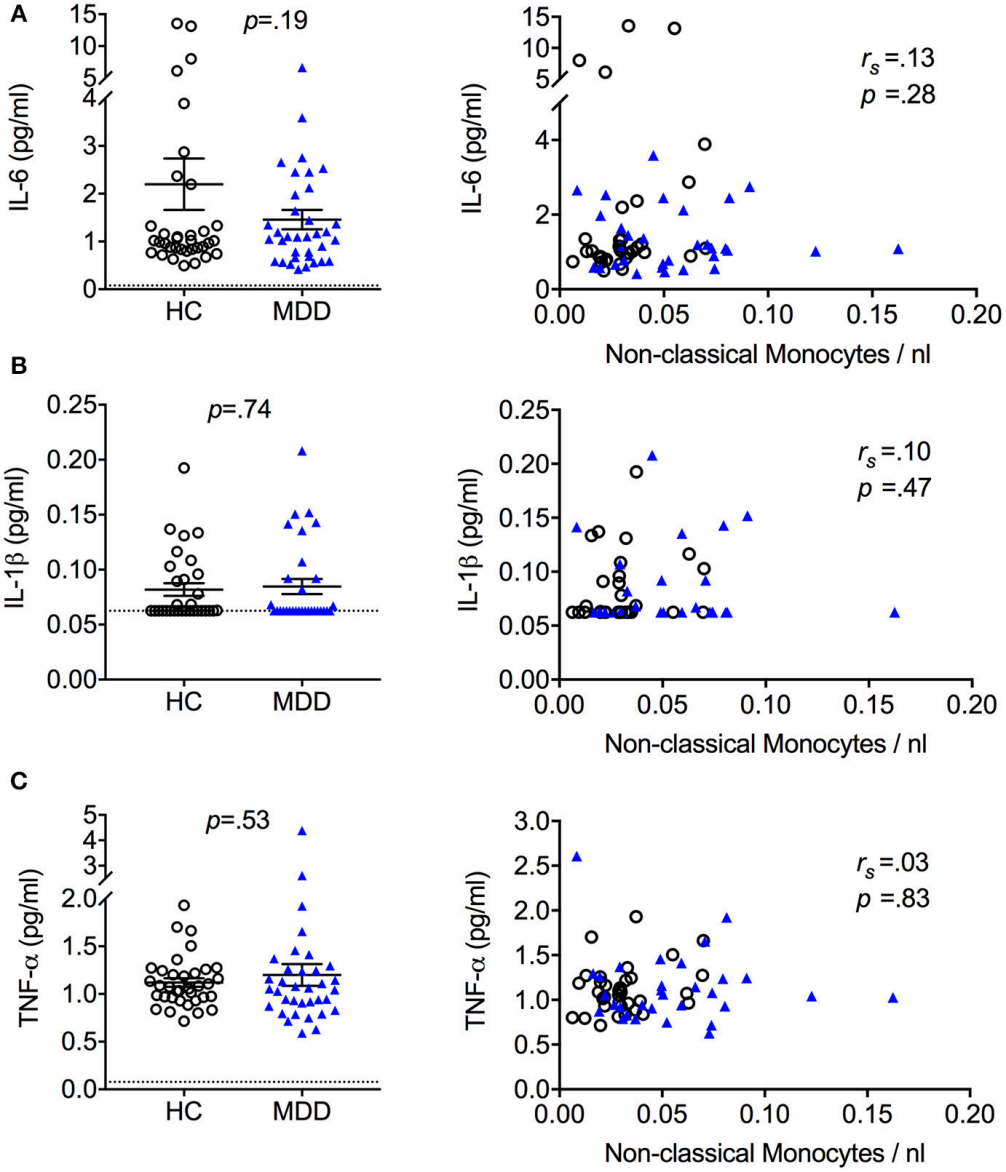
Serum cytokine levels in MDD patients and matched healthy controls (mean + S.E.M.). Serum cytokine levels of IL-6 **(A)**, IL-1β **(B)**, and TNF-α **(C)** quantified using a high sensitivity ELISA and association between serum cytokine levels and absolute cell counts of non-classical monocytes. Dotted lines represent the limit of detection 55% of IL-1β values from HC and 58% of IL-1β values from MDD patients were < 0.5 × limit of quantification (LOQ, dotted line) and thus set to 0.5 × LOQ. *r*_*s*_ = Spearman's rho.

No significant group differences were observed for high sensitivity CRP levels (MDD: 1.67 ± 1.7 mg/L; HC: 1.46 ± 1.5mg/L). Moreover, MDD patients and controls did not differ in serum levels of the cytokines IL-6, IL-1β, or TNF-α (Figure 3). There were also no significant associations between serum cytokine levels and monocyte subset counts (see Figure 3).

### Clinical correlates

To examine the relationship of steroid-related gene expression and immune phenotype with clinical variables, we ran correlation analyses. Out of 64 correlation coefficients computed, only two reached statistical significance, which is well within the range of chance findings (all *p-*values > 0.05; Table S3).

## Discussion

Our study has three main results. First, patients with MDD showed a shift toward non-classical monocytes with no group differences in major T cell subset frequencies and phenotype, B cells or NK cells. Second, expression of key steroid-signaling genes *GR* and *GILZ* was lower in monocytes obtained from MDD patients with no group differences observed in T cells. Third, monocyte phenotype and steroid receptor expression was not related to circulating levels of cortisol or circulating levels of the cytokines IL-6, IL-1β, or TNF-α.

Human monocytes can be divided into classical, intermediate and non-classical subsets that are functionally heterogeneous. Increasing evidence suggests that a higher frequency of the non-classical monocyte subtype is conducive to chronic inflammation, as seen in various illnesses (28–30). Here, we observed a significant shift among monocytes in patients with MDD. Intriguingly, non-classical monocytes are also associated with several somatic conditions that commonly co-occur in MDD patients (31), including coronary artery disease (32). Since patients in our MDD cohort were free of overt comorbid cardiovascular or metabolic disorders, our data suggest that monocyte subset shifts in MDD are not necessarily the consequence of comorbid somatic disorders but might occur independently or at least prior to such comorbidities in MDD. In contrast to our results, earlier studies in depressed patients did not find a link between depression and monocyte frequencies or phenotype (33) (34), although direct comparisons are hampered by differences in methodology (e.g., insufficient monocyte characterization) and study populations (e.g., elderly patients). Using an approach similar to ours, Suzuki et al. (35) recently reported no group differences in classical and non-classical monocytes between patients with MDD and healthy controls. In the study by Suzuki et al. (35) patients with MDD had a significantly higher BMI (close to obesity) compared to the control group. This is important because BMI can have a profound effect on immune responses, including major lymphocyte (36) and monocyte populations (37). Moreover, more than half of MDD patients in the study by Suzuki were only mildly depressed or in partial remission, while our sample consisted of patients with a well-described episode of at least moderate severity and many of our patients were currently hospitalized due to MDD. Thus, the exact relationship between comorbidities, demographic variables, clinical severity, and monocyte subsets should be explored in detail in the future to determine the dynamics of immune alterations over the course of MDD.

Our second main result was that lower expression of steroid signaling related genes (i.e., *GR* and *GILZ*) was restricted to monocytes and not observed in T cells. Thus, our findings both replicate and expand previous studies (7, 8). More specifically, they suggest that monocytes in MDD are characterized by a reduction of *GR* expression (and more downstream, *GILZ*) rather than changes in *MR* or cortisol bioconversion.

Previous findings of functional steroid resistance as obtained by mitogen-stimulated proliferation assays (13) have often been attributed to reduced GR expression, however, evidence for this in peripheral immune cells is mixed (38). To our knowledge, no study to date has assessed GR expression or other GC-related genes in T cells specifically. Studies examining functional steroid resistance have typically used proliferation assays or cytokine production in whole blood (13, 39) or PBMC culture systems (38, 40, 41). Our findings of unaltered *GR* and *GILZ* expression in T cells would suggest that functional steroid resistance in T cells as indicated by proliferation assays is unlikely to be explained by reduced steroid receptor expression in T cells but may either be due to alterations in other components of the signaling cascade or mediated indirectly via other cell populations such as monocytes.

Finally, healthy controls and the MDD group showed similar circadian HPA axis activity, CRP levels, and circulating levels of IL-6, IL-1β, or TNF-α, which did not correlate with monocyte phenotype. A similar pattern of reduced *GR* expression and sensitivity in PBMCs without HPA axis hyperactivity was found in coronary heart disease patients with depression (42). Interestingly, evidence from studies with chronically stressed caregivers also suggests that blunted steroid signaling and altered inflammatory gene expression in monocytes can occur independently of HPA axis output (43, 44). Taken together, this suggests that MDD-associated changes in monocyte phenotype and steroid signaling gene expression do not require the presence of detectable differences in cortisol or cytokines such as IL-6, IL-1β, or TNF-α.

Strengths of our study include careful matching of patients and controls, in-depth manual as well as algorithm-based characterization of lymphocytes and monocytes and cell-specific investigation of steroid-related gene expression. Yet, several limitations need to be acknowledged. For example, we did not include cell-specific functional readouts, such as cytokine production. Our study adds relevant new information by providing direct evidence for specificity of steroid-signaling gene expression within the immune system of MDD patients. However, we acknowledge that our analysis of gene expression in pan-monocytes (CD14^+^) and pan-T cells (CD3^+^) still includes heterogeneous cell populations within each lineage. Some limitations also concern the clinical characteristics of our sample. Our main concern here was to control for somatic comorbities, antidepressant medication, age, sex and BMI. This is important to limit the impact of such confounds on the variables of interest in case-control studies. However, this approach also limits generalizability to the MDD population at large. Similarly, we were likely underpowered in the exploratory analyses of clinical correlates and the non-significant results in this area should be interpreted with caution. Lastly, we did not measure several lifestyle factors such as physical activity, diet, sleep (45), that may affect cell-mediated immunity. However, given the close matching for BMI, at least major metabolic effects due to group differences in physical activity or diet appear unlikely.

In summary, our results suggest that in MDD, shifts in monocyte phenotype and altered expression of genes involved in steroid signaling, can occur in the absence of HPA axis hyperactivity or elevated levels of circulating cytokines such as IL-6, IL-1β, or TNF-α. This provides evidence for a possible divergence in steroid signaling-related gene expression between monocytes and T cells in MDD and could provide a starting point for further research into the role of monocyte subsets in major depression.

## Author contributions

SMG and CO: conception and design. HH and SG: execution of experiments. HH, AT, JN, RZ, and DP: acquisition of data. HH, SG, and KP: analysis of data. HH, SG, SMG, and CO: interpretation of data. SMG and CO: obtained funding. HH, SG, and SMG: drafting of the manuscript. AT, JN, KP, CR, FP, KW, DP, and CO: revision of the manuscript for important intellectual content.

### Conflict of interest statement

CO has received honoraria for lectures from Lundbeck and Neuraxpharm and compensation as a member of the scientific advisory board of Allergan, Lundbeck and Neuraxpharm. SMG has received honoraria for consulting from Mylan GmbH and Almirall S.A. The remaining authors declare that the research was conducted in the absence of any commercial or financial relationships that could be construed as a potential conflict of interest.
