# Supplementary material for: Pro-inflammatory Monocyte Phenotype and Cell-Specific Steroid Signaling Alterations in Unmedicated Patients With Major Depressive Disorder
Source: Front Immunol. 2018 Nov 23;9:2693. doi: 10.3389/fimmu.2018.02693 (PMC6265986; doi:10.3389/fimmu.2018.02693)
Supplement: Supplementary file 1 [file Data_Sheet_1.pdf]

Supplementary Material

"Pro-inflammatory monocyte phenotype and cell-specific steroid signaling alterations in unmedicated patients with major depressive disorder"

by Hasselmann *et al.*

TABLE S1 | Antibody Panels.

TABLE S2 | List of patients with MDD and healthy controls matched for age, sex, body mass index, and current smoking status.

TABLE S3 | Correlations between steroid signaling-related gene expression, PBMC phenotype and clinical variables in patients with MDD.

FIGURE S1 | Flow cytometry gating strategy for identification of lymphocyte phenotype.

FIGURE S2 | Flow cytometry gating strategy for identification of classical ($CD14^{++}$ $CD16^{-}$), intermediate ($CD14^{++}CD16^{+}$) and non-classical ($CD14^{+}CD16^{++}$) monocytes and exemplary staining for a patient-control pair.

FIGURE S3 | Routine laboratory blood cell counts of circulating leukocytes and proportions of monocyte and T cells subsets, as well as B cells and Nk cells as measured by flow cytometry.

FIGURE S4 | Salivary cortisol levels in patients with MDD and matched healthy controls.

TABLE S1 | Antibody Panels.

T cell phenotype and activation	Treg	Non-T cells
HLA-DR (LN3)	HLA-DR	HLA-DR
CCR7 (G043H7)	CD25 (M-A251)	CD56 (HCD56)
CD4 (RPA-T4)	CD4	CD4
CD45RA (HI100)	CD45-RA	CD20 (2H7)
CD38 (HB-7)	CD127 (A019D5)	CD16 (3G8)
Zombie NIR	Zombie NIR	Zombie NIR
CD3 (OKT3)	-	CD14 (HCD14)
CD8 (SK1)	CD3	CD3

Three different antibody panels were applied to each sample to analyze PBMC subsets: Naive, Memory and Effector T cells as well as activated T cells (T cell phenotype and activation panel), regulatory T cells (Treg) or non-T cell peripheral blood mononuclear cells including monocytes, natural killer cells and B cells (Non-T cells). Antibody clones are indicated in brackets.

TABLE S2 | List of patients with MDD and healthy controls matched for age, sex, body mass index and current smoking status. Depression severity (MADRS), psychiatric comorbidity as well as depression subtype according to DSM5 are given where applicable.

	**Age**	**Sex**	**BMI**	**Currently Smoking**	**MADRS Score**	**Psychiatric Comorbidity**	**Subtype (DSM5)**
MDD01	47	F	19.8	No	23	SP	-
HC01	42	F	19.0	No	0		
MDD02	36	M	27.3	No	24	-	-
HC02	31	M	27.5	No	0		
MDD03	22	F	21.2	No	27	SP	-
HC03	25	F	19.5	No	2		
MDD04	30	F	27.7	No	28	PD	
HC04	25	F	23.7	No	2		
MDD05	33	F	24.4	No	25	-	melancholic
HC05	28	F	22.4	No	0		
MDD06	20	F	26.6	No	30	-	melancholic
HC06	24	F	22.2	No	0		
MDD07	26	M	22.2	No	21	-	atypical
HC07	28	M	22.2	No	0		
MDD08	30	M	26.5	Yes	28	SP	melancholic
HC08	30	M	23.1	Yes	1		
MDD9	33	F	19.7	No	29	-	melancholic
HC09	33	F	23.1	No	0		
MDD10	32	F	27.7	No	26	PD	melancholic
HC10	28	F	23.5	No	1		
MDD11	24	F	26.1	Yes	25	-	melancholic
HC11	30	F	25.8	Yes	1		
MDD12	32	M	24.3	No	19	PD	melancholic
HC12	28	M	25.0	No	0		

TABLE S2 | continued

	Age	Sex	BMI	Currently Smoking	MADRS Score	Psychiatric Comorbidity	Subtype (DSM5)
MDD13	35	F	19.7	Yes	22	-	melancholic
HC13	30	F	19.1	Yes	0		
MDD14	26	M	25.0	Yes	17	-	-
HC14	24	M	21.7	Yes	0		
MDD15	53	F	26.9	No	28	-	-
HC15	55	F	25.4	No	1		
MDD16	59	M	25.4	No	25	-	melancholic
HC16	57	M	21.6	No	1		
MDD17	32	M	27.6	Yes	17	-	-
HC17	27	M	25.3	Yes	0		
MDD18	39	F	20.3	No	28	-	-
HC18	41	F	23.1	No	0		
MDD19	50	F	26.7	No	27	-	melancholic
HC19	44	F	28.5	No	0		
MDD20	19	F	21.6	Yes	19	-	-
HC20	22	F	19.7	Yes	4		
MDD21	45	F	21.9	Yes	25	-	melancholic
HC21	52	F	22.5	Yes	1		
MDD22	21	F	23.2	Yes	25	-	-
HC22	19	F	21.6	Yes	0		
MDD23	31	M	20.5	No	29	-	melancholic
HC23	30	M	22.4	No	6		
MDD24	18	M	16.8	Yes	23	-	melancholic
HC24	23	M	17.7	Yes	5		
MDD25	47	F	19.5	No	27	GAD	melancholic
HC25	49	F	22.2	No	6		

TABLE S2 | continued

	Age	Sex	BMI	Currently Smoking	MADRS Score	Psychiatric Comorbidity	Subtype (DSM5)
MDD26	26	M	18.3	Yes	32	-	melancholic
HC26	24	M	19.4	Yes	1		
MDD27	24	F	28.7	No	25	-	melancholic
HC27	28	F	26.6	No	0		
MDD28	18	F	30.4	No	19	SP	-
HC28	21	F	32.1	No	2		
MDD29	24	M	25.3	No	12	-	-
HC29	30	M	23.0	No	0		
MDD30	29	F	21.9	No	35	-	melancholic
HC30	26	F	24.8	No	5		
MDD31	25	F	21.0	No	31	PTSD	atypical
HC31	30	F	24.1	No	0		
MDD32	55	F	32.2	No	24	-	melancholic
HC32	46	F	31.9	No	0		
MDD33	27	F	25.3	Yes	31	SP & GAD	-
HC33	36	F	24.6	Yes	1		
MDD34	20	F	23.7	No	13	-	-
HC34	21	F	25.1	No	2		
MDD35	21	F	22.9	No	32	-	melancholic
HC35	22	F	21.9	No	1		

GAD: generalized anxiety disorder, PD: panic disorder, PTSD: post-traumatic stress disorder, SP: social phobia

TABLE S3 | Correlations between steroid signaling-related gene expression, PBMC phenotype and clinical variables in patients with MDD.

	BAI	BDI-II	MADRS	CTQ
**Steroid-related Gene Expression**				
T cells				
GR	-0.10	-0.297	-0.18	-0.154
	0.96	0.08	0.3	0.38
MR	0.012	-0.1	0.088	-0.224
	0.95	0.57	0.62	0.19
GILZ	0.322	0.025	0.089	-0.316
	0.06	0.89	0.61	0.06
11ß-HSD1	0.137	-0.313	-0.14	-0.041
	0.44	0.07	0.43	0.82
Monocytes				
GR	-0.186	-0.232	-0.283	-0.007
	0.28	0.18	0.1	0.97
MR	0.129	0.128	0.236	-0.207
	0.46	0.47	0.17	0.23
GILZ	0.228	0.307	0.163	-0.169
	0.19	0.07	0.35	0.33
11ß-HSD1	0.137	0.168	0.219	-0.278
	0.46	0.37	0.24	0.13
**PBMC Phenotype**				
Monocytes				
Classical Monocytes %	-0.047	-0.018	-0.014	-0.205
	0.79	0.92	0.94	0.25
Intermediate Monocytes %	0.221	0.033	-0.133	0.258
	0.22	0.86	0.46	0.15
Non-classical Monocytes '%	-0.046	0.061	0.023	0.166
	0.96	0.74	0.9	0.36

TABLE S3 | continued

	BAI	BDI-II	MADRS	CTQ
Lymphocytes				
$CD4^+$ T Cells	-0.009	0.203	0.035	-0.286
	0.96	0.26	0.85	0.11
$CD8^+$ T Cells	0.043	-0.161	-0.112	0.383
	0.81	0.37	0.54	0.03
Regulatory T Cells	-0.083	-0.228	-0.332	-0.13
	0.67	0.24	0.08	0.5
Cytotoxic Natural Killer Cells	0.215	-0.102	-0.023	-0.034
	0.23	0.57	0.9	0.85
Regulatory Natural Killer Cells	-0.075	0.02	-0.371	0.324
	0.68	0.91	0.03	0.07
B cells	-0.147	-0.282	-0.212	-0.03
	0.41	0.11	0.24	0.86

BAI: Beck Anxiety Inventory, BDI-II: Beck Depression Inventory II, CTQ: Childhood Trauma Questionnaire, MADRS: Montgomery Asberg Depression Rating Scale. All correlation coefficients denote Spearman’s rho with respective p values (two-tailed) indicated below.

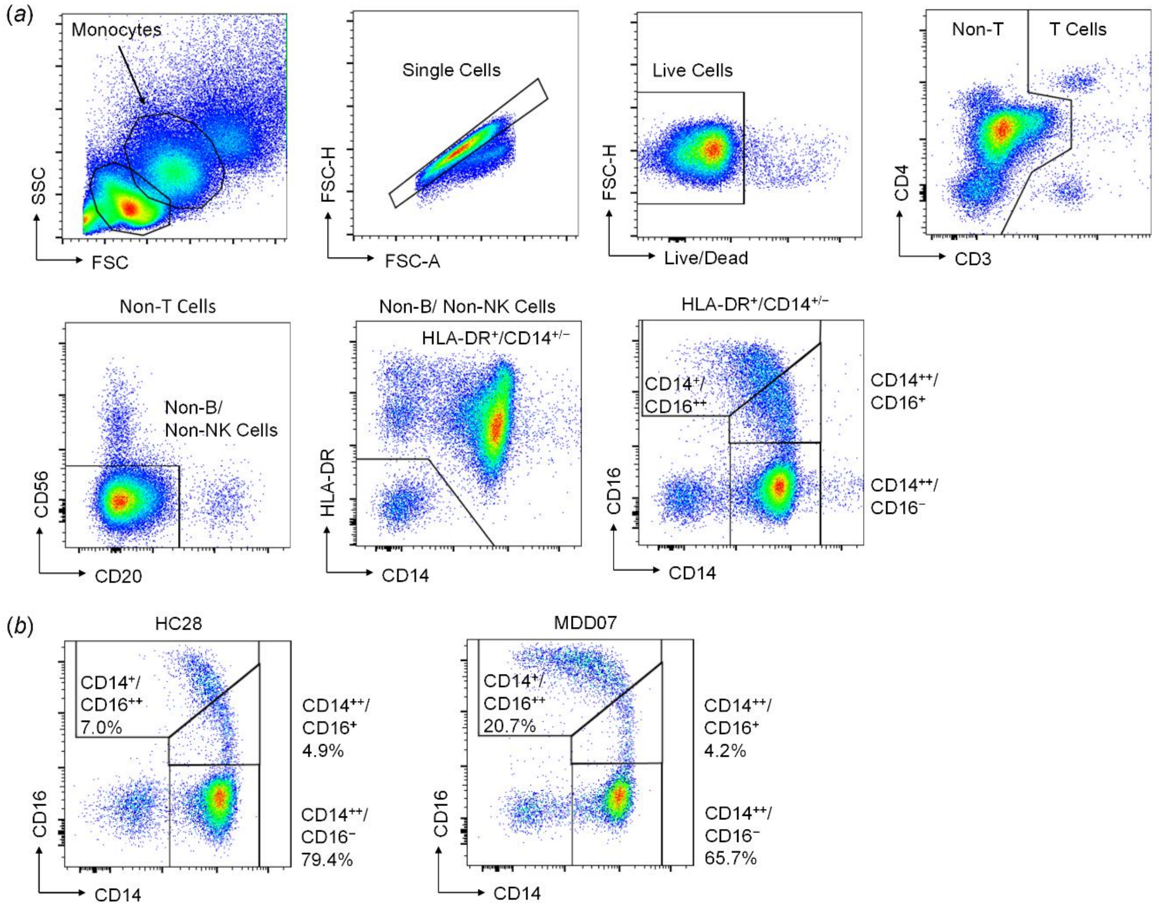

FIGURE S1 | Flow cytometry gating strategy for identification of classical ($CD14^{++}$ $CD16^{-}$), intermediate ($CD14^{++}CD16^{+}$) and non-classical ($CD14^{+}CD16^{++}$) monocytes and exemplary staining for a patient-control pair. **(*a*)** Monocytes were identified by forward (FSC) and sideward scatter (SSC) properties. Next, doublets and dead cells were excluded as were remaining $CD3^{+}$ T cells, B cells ($CD20^{+}$), natural killer cells ($CD56^{+}$) and $CD14^{-}$/HLA-DR^{-} cells. Classification of monocytes based on surface expression of CD14 and CD16 followed established guidelines (21). **(*b*)** Exemplary monocyte staining in a representative patient-control pair.

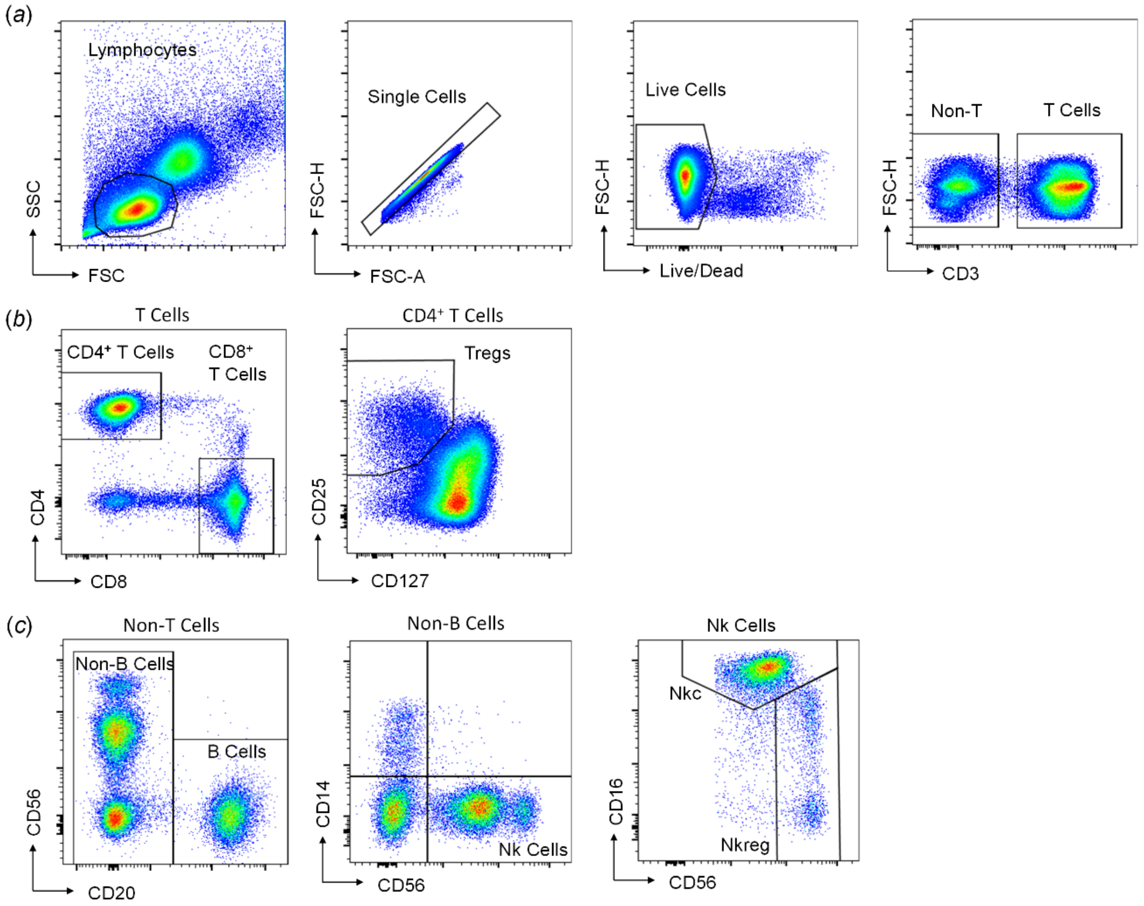

FIGURE S2 | Flow cytometry gating strategy for identification of lymphocyte phenotype. **(*a*)** First, lymphocytes were identified by forward (FSC) and sideward scatter (SSC) properties. Next, doublets and dead cells were excluded. Next, cells were gated for CD3 positivity (T cells) or negativity (Non-T cells). **(*b*)** T cells were further divided into $CD4^+$ helper and $CD8^+$ cytotoxic T cells. Among $CD4^+$ T cells, regulatory T cells were identified as $CD25^+CD127^-$. **(*c*)** Among non-T cells, $CD20^+$ B cells were identified. Next, among $CD20^-$ cells NK cells were selected for CD56 positivity and CD14 negativity. Lastly, cytotoxic NK cells (Nkc) were defined as $CD56^+CD16^+$ and NK cells with a putatively regulatory phenotype (Nkreg) as $CD56^+CD16^{dim/-}$ as suggested by Maecker *et al.* 2012.

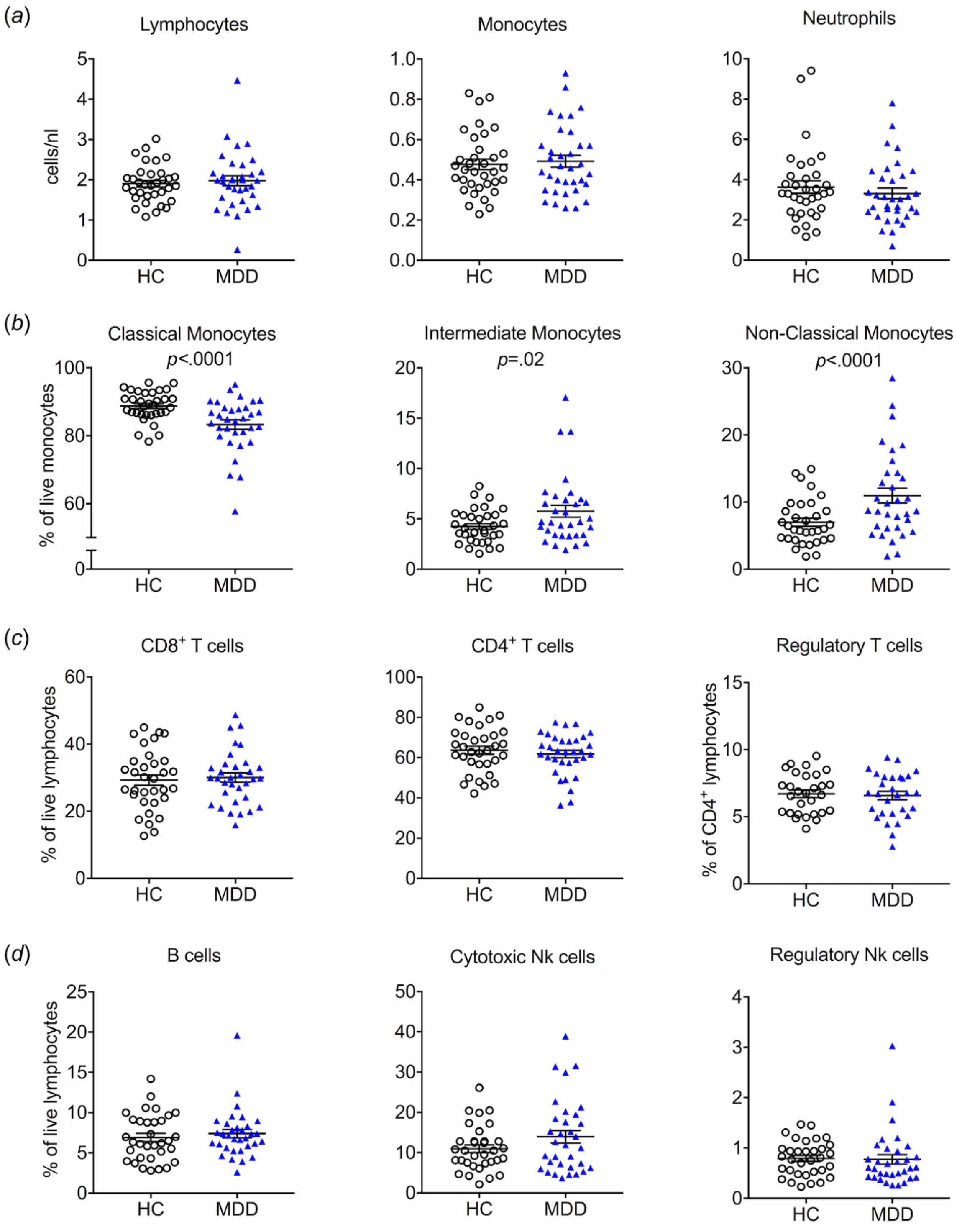

FIGURE S3 | ***(a)*** Routine laboratory blood cell counts of circulating leukocytes and proportions of ***(b)*** monocyte and ***(c)*** T cells subsets, as well as ***(d)*** B cells and Nk cells as measured by flow cytometry (mean ± S.E.M.). If not depicted, all *p*-values > 0.1.

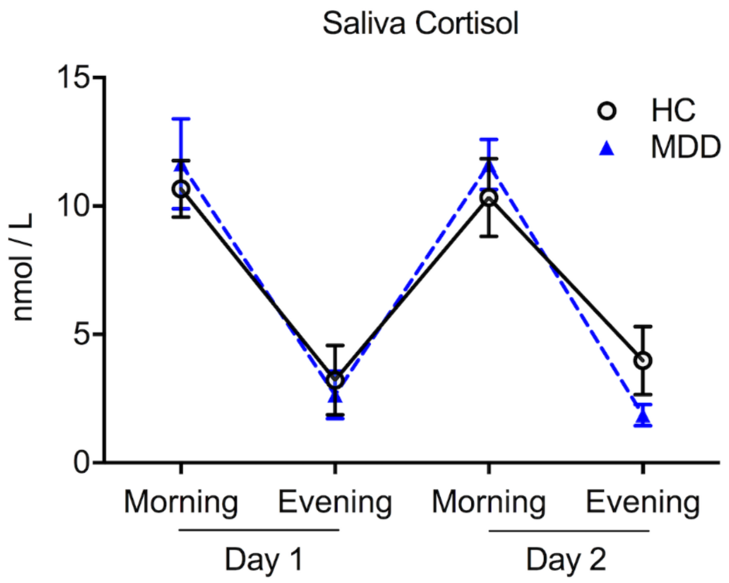

FIGURE S4 | Salivary cortisol levels in patients with MDD and matched healthy controls (mean ± S.E.M). Circadian HPA axis activity was estimated by salivary cortisol measures (8:00 a.m. and 22:00 p.m.) collected on two consecutive days (day 1 n = 28, day 2 n = 29).
